# Enhancement of Antioxidant Mechanisms and Reduction of Oxidative Stress in Chickens after the Administration of Drinking Water Enriched with Polyphenolic Powder from Olive Mill Waste Waters

**DOI:** 10.1155/2017/8273160

**Published:** 2017-08-24

**Authors:** Aliki Papadopoulou, Konstantinos Petrotos, Dimitrios Stagos, Konstantinos Gerasopoulos, Antonios Maimaris, Haralampos Makris, Ioannis Kafantaris, Sotiria Makri, Efthalia Kerasioti, Maria Halabalaki, Vincent Brieudes, Georgia Ntasi, Stylianos Kokkas, Pavlos Tzimas, Panagiotis Goulas, Alexander M. Zakharenko, Kirill S. Golokhvast, Aristidis Tsatsakis, Demetrios Kouretas

**Affiliations:** ^1^Department of Biochemistry and Biotechnology, University of Thessaly, Viopolis, 41500 Larissa, Greece; ^2^Department of Biosystems Engineering, Technical Education Institute of Thessaly, 41110 Larissa, Greece; ^3^Department of Animal Production, Technical Education Institute of Thessaly, 41110 Larissa, Greece; ^4^Department of Pharmacognosy and Natural Products Chemistry, Faculty of Pharmacy, University of Athens, Panepistimiopolis Zografou, 15771 Athens, Greece; ^5^PharmaGnose S.A., Papathanasiou 24, 34100 Eyboia, Greece; ^6^Department of Agricultural Engineering Technologists, Technical Education Institute of Thessaly, 41110 Larissa, Greece; ^7^Scientific Educational Centre of Nanotechnology, Far Eastern Federal University, Engineering School, 10 Pushkinskaya Street, 690950 Vladivostok, Russia; ^8^Laboratory of Toxicology, School of Medicine, University of Crete, 71003 Heraklion, Greece

## Abstract

The aim of the study was to examine the effects of a polyphenolic powder from olive mill wastewater (OMWW) administered through drinking water, on chickens' redox status. Thus, 75 chickens were divided into three groups. Group A was given just drinking water, while groups B and C were given drinking water containing 20 and 50 *μ*g/ml of polyphenols, respectively, for 45 days. The antioxidant effects of the polyphenolic powder were assessed by measuring oxidative stress biomarkers in blood after 25 and 45 days of treatment. These markers were total antioxidant capacity (TAC), protein carbonyls (CARB), thiobarbituric acid reactive species (TBARS) and superoxide dismutase activity (SOD) in plasma, and glutathione (GSH) and catalase activity in erythrocytes. The results showed that CARB and TBARS were decreased significantly in groups B and C, and SOD decreased in group B compared to that in group A. TAC was increased significantly in group C and GSH was increased in group B, while catalase activity was increased in groups B and C compared to that in group A. In conclusion, this is the first study showing that supplementation of chickens with polyphenols from OMWW through drinking water enhanced their antioxidant mechanisms and reduced oxidative stress-induced damage.

## 1. Introduction

Free radicals are atoms, molecules, or ions that have unpaired valence electrons [1]. Free radicals such as reactive oxygen species (ROS) are produced in living organisms either from normal essential metabolic processes or from external sources (e.g., exposure to X-rays, air pollutants, and industrial chemicals) [2]. Due to the unpaired electrons, free radicals are very reactive species and their overproduction can cause damage to all biological macromolecules such as DNA, proteins, and lipids, thus resulting in cell damage and subsequently in manifestation of pathological conditions [1]. Oxidative stress is defined as an imbalance between the production of free radicals and the ability of the organism to detoxify them or counteract their harmful effects through neutralization by antioxidants and is responsible for the cause of several diseases [1, 2]. Several studies have suggested that oxidative stress in farm animals may be involved in pathological conditions affecting animal production and welfare [3]. For example, the hot and humid environment in aviaries may cause heat-induced oxidative stress in chickens, which in turn reduces growth and meat quality [4]. Thus, administration of natural antioxidant compounds to chickens has been proposed as a means for reducing the oxidative stress-induced adverse effects [5–7]. Polyphenols are bioactive phytochemical compounds and mostly studied due to their antioxidant properties. Polyphenols are secondary metabolites and act defensively in plants against pathogens and UV-mediated stress [8]. They are divided mainly into four groups according to their chemical structure, flavonoids, phenolic acids, stilbenes, and lignans [8]. Many studies have suggested that polyphenols' antioxidant activity may improve the well-being of living organisms and protect against several diseases [9–11]. Moreover, polyphenol consumption causes lower toxicity and fewer side effects than other chemical compounds used for prevention from diseases [12].

One of the polyphenols' sources is olive oil obtained from olive tree fruit (*Olea europaea* L.). The main polyphenolic compounds found in olive oil are tyrosol, hydroxytyrosol, oleuropein, and pinoresinol, exhibiting potent antioxidant properties [13, 14]. For example, olive oil-supplemented diet has been shown to protect chicken skeletal muscle from heat stress-induced oxidative stress [15]. Moreover, byproducts of olive oil production such as olive mill wastewater (OMWW) contain polyphenols (e.g., p-coumaric acid, homovanillic acid, caffeic acid, protocatechuic acid, 3,4-dihydroxymandelic acid, vanillic acid, and ferulic acid) with antioxidant activity [16–18]. In previous studies, we have demonstrated that administration of feed supplemented with polyphenols from OMWW improved the redox status in chickens and pigs [7, 19]. However, polyphenols from OMWW byproducts have not been so far administered to chickens through water supply. Polyphenols' supplementation through water or feed may affect differently their absorption and consequently their bioavailability and bioactivities' potency. Thus, in the present study, water-diluted polyphenolic powder from OMWW was administered to broiler chickens. Then, the possible enhancement of antioxidant mechanisms or the protection of macromolecules from ROS-induced damage was assessed by measuring oxidative stress biomarkers in broilers' blood.

## 2. Materials and Methods

### 2.1. Polyphenolic Powder Description

The product with the name MEDOLIVA® is produced according to an established patented procedure (patent application number: 20120100569—Greek Industrial Property Organization), for obtaining polyphenols from OMWW based on the use of ceramic membrane microfiltration using clean vegetable waters from olive mills. The product comes in a liquid form which is stable and safe without the use of conservatives. The polyphenolic liquid product is transformed into powder, using maltodextrin as nanoencapsulation material, through the freeze dryer technology.

### 2.2. HPLC Analysis for the Identification of Polyphenols of Medoliva Powder

All HPLC analyses were carried out on a Hitachi Co-Japan system (Japan) equipped with a quaternary pump L-2130, column thermostat L-2300, and diode array L-2455 detector. The column used was a Pinnacle II RP C18 (150 mm × 4.6 mm) with a guard column of Kromasil 100-5 C18 (3.0 × 4.6 mm). Injection was by means of a Hitachi Elite LaChrom Autosampler L-2200 with a 20 *μ*l fixed loop. For the chromatographic analyses, HPLC grade water was used, whereas all HPLC solvents were filtered prior to use through cellulose acetate membranes of 0.45 *μ*M pore size. Chromatographic data were acquired and processed using Agilent EZChrom Elite software (Agilent, CA, USA).

For the preparation of the sample analyzed by HPLC, 10 ml of the sample solution was extracted four times with HPLC grade ethyl acetate, and then the solvent was evaporated and the remaining organic phase was dissolved in 4 ml of HPLC grade methanol and collected to HPLC type glass bottles for further analysis.

The HPLC analysis was carried out at 40°C (maintained by the column thermostat) using samples of 20 *μ*l, which were directly injected by means of a Hitachi Elite LaChrom Autosampler L-2200. The gradient eluted consisted of solvent A [obtained by the addition of 3% acetic acid in 20 mM sodium acetate aqueous solution, pH 3.2] and solvent B (acetonitrile, CH_3_CN). Run time was set at 28 min with a constant flow rate at 1.0 ml/min in accordance with the following gradient time table: at zero time, 100% A; after 3 min, the pumps were adjusted to 88% A and 12% B; at 10 min, 79% A and 21% B; at 12 min, 61% A and 39% B; at 18 min, 46% A and 54% B; at 25 min, 40% A and 60% B; and finally, at 28 min, 100% B. The analysis was monitored at 280 nm for oleuropein, hydroxytyrosol, and tyrosol and at 355 nm for flavonols simultaneously. Three replicate experiments were carried out for each sample examined. Peaks were identified by comparing their retention time and UV-vis spectra with the reference compounds, and data were quantitated using the corresponding curves of the reference compounds as standards. All standards were dissolved in methanol.

### 2.3. Assessment of the Total Polyphenolic Content (TPC)

The TPC of the Medoliva powder was determined in accordance with a modified version of the Folin-Ciocalteu method [20].

Initially, 1 gr of powder was added to 20 ml of extraction solution (80% *v*/*v* ethanol, 20% distilled water containing 1% HCl). The mixture was added to a 50 ml flask and centrifuged at 3000 rpm for 20 min. Then, the supernatant solution was added to a 50 ml volumetric flask that was filled with distilled water until the final volume. Afterwards, 5 ml of the solution was transferred to a volumetric flask of 25 ml that was filled with water until the final volume.

After the above preparation, 1.6 ml of sample was added to a tube along with 0.3 ml of 20% Na_2_CO_3_ deionized water and 0.1 ml of Folin-Ciocalteu reagent. The mixture was allowed to stand at room temperature for 2 h. Absorbance was measured at 725 nm versus a blank. The results are expressed as gallic acid equivalents using the standard curve (absorbance versus concentration) prepared from authentic gallic acid.

### 2.4. Animals

The experiment was reviewed and approved by the institutional review board and the appropriate state authority. Seventy-five broiler chickens (Hubbard-Sasso hybrid), 15 days old, were purchased from “Bloutsos” aviary (Trikala, Greece). Chickens were housed under controlled environmental conditions (12-hour light/dark cycle, temperature 18–21°C, and humidity 50–70%). Then, they were randomly divided into three experimental groups (25 chickens per group) as follows: group A, chickens were given fresh tap water without polyphenolic powder; group B, chickens were given polyphenols dissolved in water at a concentration of 200 *μ*g/ml powder (equals to 20 *μ*g/ml of polyphenols); and group C, chickens were given polyphenols dissolved in water at a concentration of 500 *μ*g/ml powder (equal to 50 *μ*g/ml of polyphenols). The Medoliva powder was instantly soluble in the water, as it was prepared by freeze drying. The concentrations of 200 and 500 *μ*g/ml did not cause a solubility problem. The addition of the powder to the water was made at a daily basis, and for this reason, there was no problem of stability. Chickens' weight was monitored every five days throughout the 45 days of the experiment. Moreover, feed and water consumption were recorded at a daily basis.

### 2.5. Blood Collection

Blood samples were drawn at the age of 40 days (i.e., after 25 days of treatment) and 60 days (i.e., after 45 days of treatment). 4 ml of blood was collected from the brachial vein of each chicken and placed into 5 ml aseptic EDTA tubes. Blood samples were centrifuged immediately at 1370*g* for 10 min at 4°C, and the plasma was collected and used for measuring total antioxidant capacity (TAC), thiobarbituric acid reactive species (TBARS), and protein carbonyls (CARB). The packed erythrocytes were lysed with distilled water (1 : 1 *v*/*v*), inverted vigorously, and centrifuged at 4020*g* for 15 min at 4°C, and the erythrocyte lysate was collected for the measurement of reduced glutathione (GSH) and catalase activity.

### 2.6. Oxidative Stress Biomarkers

Glutathione (GSH) was measured according to the method of [21]. In particular, 20 *μ*l of erythrocyte lysate, treated with 5% trichloroacetic acid (TCA), was mixed with 660 *μ*l of 67 mM sodium potassium phosphate (pH 8.0) and 330 *μ*l of 1 mM 5,5-dithiobis-2-nitrobenzoate (DTNB). The samples were incubated in the dark at room temperature for 45 min, and the absorbance was read at 412 nm. GSH concentration was calculated on the basis of a calibration curve made using commercial standards.

Catalase activity was determined using the method of [22]. Briefly, 4 *μ*l of erythrocyte lysate (diluted 1 : 10) was added to 2991 *μ*l of 67 mM sodium potassium phosphate (pH 7.4), and the samples were incubated at 37°C for 10 min. A total of 5 *μ*l of 30% hydrogen peroxide was added to the samples, and the change in absorbance was immediately read at 240 nm for 1.5 min. Calculation of catalase activity was based on the molar extinction coefficient of H_2_O_2_.

The determination of superoxide dismutase (SOD) activity was based on the method of nitroblue tetrazolium salt (NBT) according to Oberley and Spitz [23]. More specifically, this assay included a negative control made by mixing 800 *μ*l of SOD buffer [1 mM diethylenetriaminepentaacetic acid (DETAPAC) in 0.05 M potassium phosphate buffer (pH 7.8); 1 U catalase; 5.6 × 10^−5^ M NBT; 10^−4^ M xanthine] with 100 *μ*l of 0.05 M potassium phosphate buffer. Subsequently, ~60 mU of xanthine oxidase (XO) was added and the rate of increase in absorbance was measured at 560 nm for 3.5 min. In the test samples, 100 *μ*l of plasma was added to 800 *μ*l of SOD buffer followed by the addition of ~60 mU of XO and the rate of increase in absorbance was measured for 3.5 min at 560 nm. Calculation of SOD activity in the test samples was based on the percent inhibition of the rate of increase in absorbance. The rate of increase in absorbance (A) per minute for the negative control and for the tested samples was determined by formula (1), and the percentage inhibition for each sample was calculated using formula (2):

(1) $$\begin{array}{c} \frac{\Delta A_{560\;nm}}{min}=\frac{A_{560\;nm}final-A_{560\;nm}initial}{3.5\;min}, \end{array}$$

(2) $$\begin{array}{c} \%\;Inhibition=\left[\frac{\Delta \left(\left(A_{560\;nm}\right)/\left({min}_{negative\;control}\right)\right)-\Delta \left(\left(A_{560\;nm}\right)/\left({min}_{sample}\right)\right)}{\Delta \left(\left(A_{560\;nm}\right)/\left({min}_{negative\;control}\right)\right)}\right]\times 100. \end{array}$$

The determination of TAC was based on the method of [24]. Briefly, 20 *μ*l of plasma was added to 480 *μ*l of 10 mM sodium potassium phosphate (pH 7.4) and 500 *μ*l of 0.1 mM 2,2-diphenyl-1-picrylhydrazyl (DPPH) free radical, and the samples were incubated in the dark for 30 min at room temperature. The samples were centrifuged for 3 min at 20,000*g*, and the absorbance was read at 520 nm. TAC is presented as mmol of DPPH reduced to 2,2-diphenyl-1-picrylhydrazine (DPPH : H) by the antioxidants of plasma.

For the determination of TBARS, a slightly modified assay of [25] was used. According to this method, 100 *μ*l of plasma was mixed with 500 *μ*l of 35% TCA and 500 *μ*l of Tris-HCl (200 mmol/L; pH 7.4) and incubated for 10 min at room temperature. 1 ml Na_2_SO_4_—thiobarbituric acid (TBA) solution—was added, and the samples were incubated at 95°C for 45 min. The samples were cooled on ice for 5 min and were vortexed after 1 ml of 70% TCA was added. The samples were centrifuged at 15,000*g* for 3 min, and the absorbance of the supernatant was read at 530 nm. A baseline shift in absorbance was taken into account by running a blank along with all samples during the measurement. Calculation of TBARS concentration was based on the molar extinction coefficient of malondialdehyde.

CARB were determined based on the method of [26]. In this assay, 50 *μ*l of 20% TCA was added to 50 *μ*l of plasma. This mixture was incubated in an ice bath for 15 min and centrifuged at 15,000*g* for 5 min at 4°C. The supernatant was discarded, and 500 *μ*l of 14 mM 2,4-dinitrophenyl hydrazine (DNPH) dissolved in 2.5 N HCl for the sample or 500 *μ*l of 2.5 N HCl for the blank was added in the pellet. The samples were incubated in the dark at room temperature for 1 h, with intermittent vortexing every 15 min, and were centrifuged at 15,000*g* for 5 min at 4°C. The supernatant was discarded, and 1 ml of 10% TCA was added, vortexed, and centrifuged at 15,000*g* for 5 min at 4°C. The supernatant was discarded, and 1 ml of ethanol-ethyl acetate (1 : 1 *v*/*v*) was added, vortexed, and centrifuged at 15,000*g* for 5 min at 4°C. This washing step was repeated twice. The supernatant was discarded, and 1 ml of 5 M urea (pH 2.3) was added, vortexed, and incubated at 37°C for 15 min. The samples were centrifuged at 15,000*g* for 3 min at 4°C, and the absorbance was read at 375 nm. Calculation of CARB concentration was based on the molar extinction coefficient of DNPH. Total plasma protein was assayed using a Bradford reagent (Sigma-Aldrich Ltd.).

### 2.7. Determination of Hydroxytyrosol in Chickens' Plasma by Mass Spectrometry

For all plasma samples, a preparation was carried out before the measurement of hydroxytyrosol. Briefly, 100 *μ*l of plasma was thawed and 480 *μ*l of acetonitrile, 60 *μ*l of methanol, and 60 *μ*l of purified water were added. Subsequently, the blurred sample due to protein precipitation was centrifuged at 12000 rpm for 10 min, and the supernatants were evaporated to dryness. Finally, with 100 *μ*l of methanol/water 1 : 1, the samples were reconstituted and at first were subjected to chromatographic separation and then analyzed at UPLC-TQD-MS/MS.

For chromatographic separation, ultra high-performance liquid chromatography system (EVOQ™, Bruker, Bremen) was employed. Mobile phases consisted of (A) deionized water with 0.1% formic acid and (B) acetonitrile (LC-MS grade). The samples (5 *μ*l) were injected to a Waters HSS (2.1 × 100 mm, 1.8 *μ*m) analytical column with 95.0% mobile phase B at a flow rate of 0.4 ml/min for 2 min. The mobile phase composition was increased to 10% B and held for 6 min before returning to 95% B for other 3.0 min to reequilibrate. Total run time injection-to-injection was 11 min. Column oven temperature was maintained at 40°C throughout.

After chromatographic separation, the eluate was directed into EVOQ triple quadrupole mass spectrometer. The mass spectrometer was operated in the negative electrospray ionization mode, the spray voltage was maintained at 4000 V, and the cone temperature was 250°C, although the heated probe temperature was 300°C. The probe and the nebulizer gas flow (nitrogen) were 30 arbs and 50 arbs, respectively. The transition of hydroxytyrosol (153.10 to 123.10 *m*/*z*) was monitored in the multiple reaction monitoring (MRM) mode with a scan time of 50 ms with collision energy of 12 eV. The calibration curve of hydroxytyrosol used for its determination consisted of six points (0.1, 0.5, 1.0, 5.0, 10.0, and 50.0 ng/ml).

### 2.8. Statistical Analysis

Data were analyzed by one-way ANOVA. The level of statistical significance was set at *p* < 0.05. All results are expressed as mean ± SD. Data were analyzed using SPSS, version 13.0 (SPSS Inc., Chicago, Il).

## 3. Results

### 3.1. Total Polyphenolic Content and Composition of Medoliva Powder

**Figure 1:**
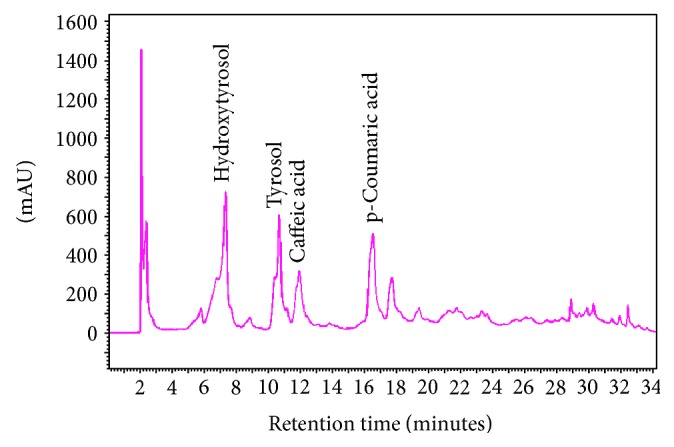
HPLC polyphenolic profile of Medoliva powder.

The TPC of the Melidova powder was 100 mg GAE/g powder (Table 1). In Figure 1, the HPLC of the polyphenolic profile of Medoliva powder is presented. From the polyphenols used as standards, four polyphenols were identified, hydroxytyrosol, tyrosol, caffeic acid, and p-coumaric acid, and their quantities were 0.50, 0.55, 0.02, and 0.04 mg/g of Medoliva powder, respectively (Table 1).

### 3.2. Assessment of Chickens' Weight

**Figure 2:**
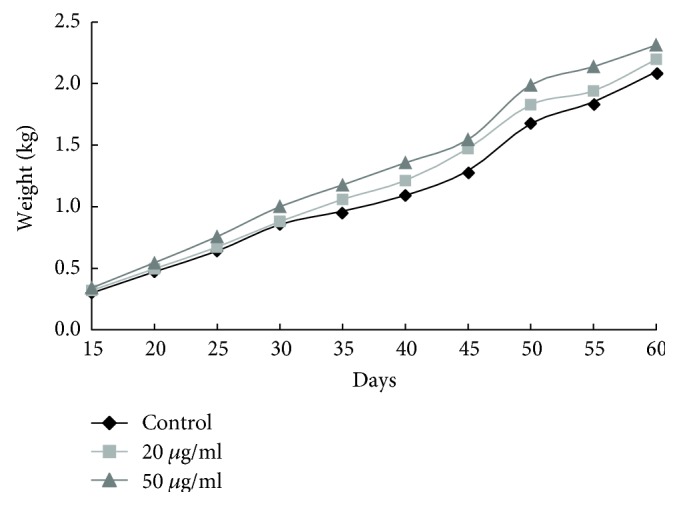
Chicken weight in relation to the days after birth.

Chickens' weight was monitored throughout the experiment. Groups B and C showed an increase in weight compared to the control group, but it was not statistically significant (Figure 2). In addition, there were not significant differences in feed consumption between the different groups (data not shown). Likewise, water consumption did not differ significantly between the different groups (Table 2).

### 3.3. Assessment of Oxidative Stress Markers in Chickens' Blood

**Figure 3:**
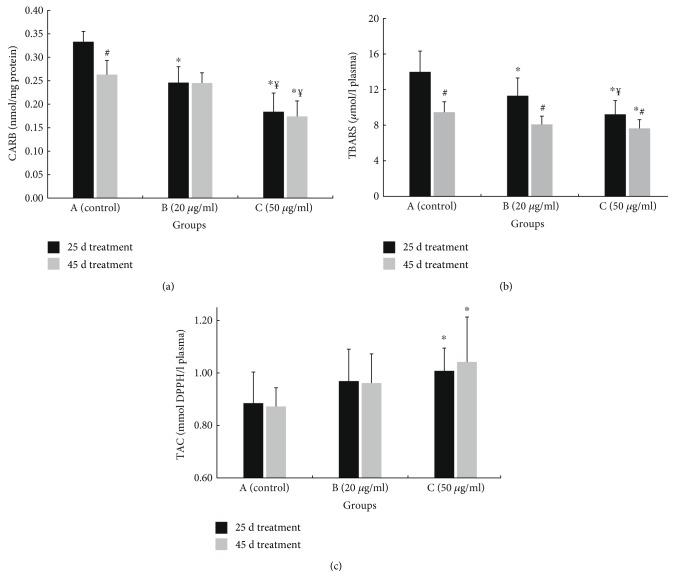
Effects on oxidative stress markers: (a) CARB, (b) TBARS, and (c) TAC, in the plasma of chickens after treatment for 25 and 45 days with water (group A; control) or water containing polyphenols at 20 *μ*g/ml (group B) or at 50 *μ*g/ml (group C). ^∗^Significantly different from the value of the control group at the same sampling time (*p* < 0.05). ^#^Significant differences between the values of the same group, measured at different sampling times (*p* < 0.05). ^¥^Significant differences between the values of Β and C groups, measured at the same sampling time (*p* < 0.05).

Regarding oxidative stress markers' measurements, all of them showed that polyphenolic powder administration through water supply improved the redox status of the broiler chickens. Specifically, CARB levels were decreased significantly in groups B and C, compared to the control group (Figure 3(a)). Group C exhibited the highest decrease in CARB levels by 44.7 and 33.8% at days 25 and 45, respectively (Figure 3(a)). In group B, there was a decrease in protein carbonyl levels by 26.1% at 25 days (Figure 3(a)). Moreover, the decrease in CARB levels was dose dependent, since there was significant reduction of CARB in group C (high dose) at both 25 and 45 days compared to group B (low dose) (Figure 3(a)).

Like protein carbonyls, TBARS levels in plasma were decreased significantly in groups B and C, compared to the control group (Figure 3(b)). Group C exhibited the greatest decrease in TBARS levels by 34.1 and 19.4%, at 25 and 45 days of treatment, respectively. In group B, TBARS were decreased by 19.1% at 25 days (Figure 3(b)). Moreover, at 25 days of treatment, there was a dose-dependent decrease in TBARS, since they were significantly lower in group C (high dose) compared to group B (low dose) (Figure 3(b)). Also, there was a time-dependent effect of the administration of polyphenols from OMWW, since in both groups B and C, TBARS were significantly lower at 45 days compared to 25 days (Figure 3(b)).

TAC in plasma was increased significantly in group C by 13.9 and 19.5% at 25 and 45 days of treatment, respectively (Figure 3(c)).

**Figure 4:**
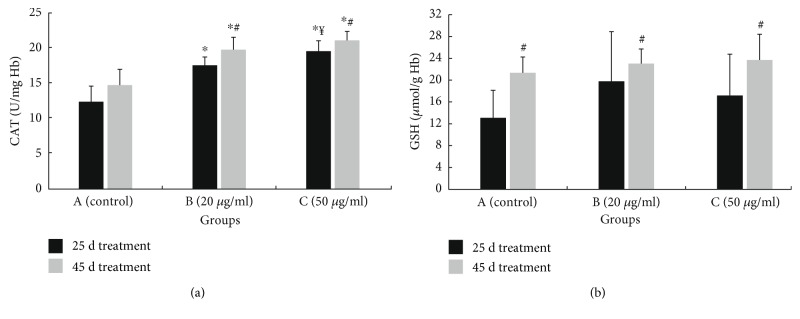
Effects on oxidative stress markers: (a) catalase activity (CAT) and (b) GSH in erythrocytes of chickens after treatment for 25 and 45 days with water (group A; control) or water containing polyphenols at 20 *μ*g/ml (group B) or at 50 *μ*g/ml (group C). ^∗^Significantly different from the value of the control group at the same sampling time (*p* < 0.05). ^#^Significant differences between the values of the same group, measured at different sampling times (*p* < 0.05). ^¥^Significant differences between the values of Β and C groups, measured at the same sampling time (*p* < 0.05).

There was also a significant increase in GSH levels in erythrocytes in group B, where GSH levels were increased by 50.9% at 25 days (Figure 4(b)). Moreover, GSH levels were increased time dependently in both B and C groups (Figure 4(b)).

Furthermore, the administration of polyphenolic powder increased catalase activity in erythrocytes. Group C demonstrated the highest increase in catalase activity by 38.4 and 30.2% at days 25 and 45, respectively (Figure 4(a)). In group B, the catalase activity was increased by 27.6 and 24.0% at days 25 and 45, respectively (Figure 4(a)). In addition, in both groups B and C, polyphenols from OMWW time dependently increased catalase activity (Figure 4(a)). There was also a dose-dependent increase of catalase activity at 25 days (Figure 4(a)).

**Figure 5:**
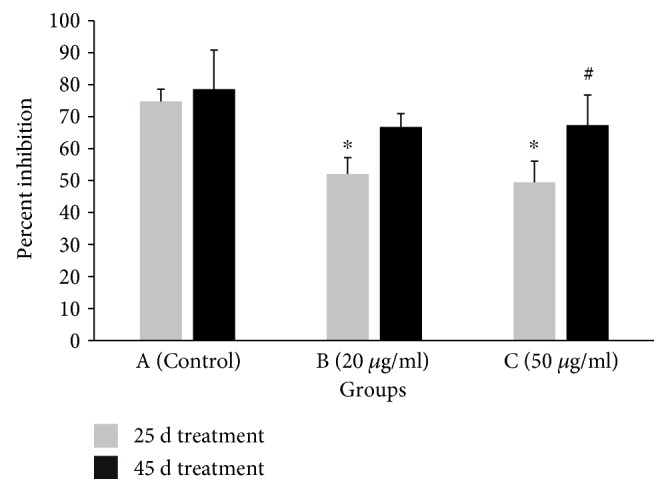
Nitroblue tetrazolium salt (NBT) assay of superoxide dismutase (SOD) activity in chicken plasma. Percent inhibition of superoxide production from 100 *μ*l of chicken plasma after treatment for 25 and 45 days with water (group A; control) or water containing polyphenols at 20 *μ*g/ml (group B) or at 50 *μ*g/ml (group C). Percent inhibition indicates the amount by which the plasma samples inhibited NBT reduction relative to a nonplasma containing control reaction. ^∗^Significantly different from the value of the control group at the same sampling time (*p* < 0.005). ^#^Significant differences between the values of the same group, measured at different sampling times (*p* < 0.05).

Regarding SOD activity in plasma, at 25 days of treatment, the enzyme activity was decreased significantly by 30.4 and 33.8% in B and C groups, respectively, compared to that in the control (Figure 5), while, at 45 days of treatment, there were not significant differences in SOD activity between the control and treatment groups (Figure 5). Moreover, in group C, SOD activity was lower at 25 days of treatment by 26.5% compared to that at 45 days (Figure 5).

Finally, it was interesting that in the control group, the chickens at 25 days of treatment (i.e., 40 days post birth) had significantly higher CARB and TBARS levels and lower GSH levels than the chickens at 45 days of treatment (i.e., 60 days post birth) (Figures 3(a), 3(b), and 4(b)).

### 3.4. Assessment of Hydroxytyrosol in Chickens' Plasma

The mass spectrometry analysis showed that, in group C, the hydroxytyrosol levels were 349.5 pg/ml plasma (Table 3). In group B, the concentration of hydroxytyrosol was below the lower limit of quantitation (100 pg/ml), while as expected, in the control group, it was not detected (lower limit of detection was 30 pg/ml) (Table 3).

## 4. Discussion

In a previous study, we have shown that supplementation of feed with polyphenols from OMWW enhanced the antioxidant mechanisms and decreased oxidative stress-induced damage in broiler chickens [7]. As known, oxidative stress may be the etiological factor for several diseases in farm animals [3]. Thus, the aim of the present study was to administer polyphenols from OMWW through water supply to chickens, as an easier way than administration through feed. For example, in order to increase the time storage and to improve the bioavailability of feed supplemented with OMWW, silage corn should be made, a laborious and time-consuming process [7]. However, the preparation for the supplementation of polyphenolic powder from OMWW through water requires only its dilution. The effects of water supplied with polyphenols from OMMW on the chickens' redox status were assessed by measuring oxidative stress markers in blood.

The results showed that the administration of water supplied with polyphenols from OMWW enhanced the antioxidant mechanisms in chickens. Specifically, TAC, an indicator of the total antioxidant capacity, was increased in the plasma of the chicken group given water supplied with OMWW, compared to that of the control. Especially, there was a significant increase in TAC after the administration of the high dose (i.e., 50 *μ*g/ml of polyphenols) of OMWW for both 25 and 45 days treatment. Interestingly, hydroxytyrosol, a major polyphenol found in OMWW, has been shown to increase nuclear factor (erythroid-derived 2)-like2 (Nrf2) expression and nuclear translocation, where it stimulated the transcription of antioxidant and detoxifying enzymes in the mouse heart [27].

The abovementioned increase in TAC could be attributed, at least in part, to the OMWW-induced increase of antioxidant molecules such as catalase enzyme activity in erythrocytes. Catalase catalyzes the decomposition of hydrogen peroxide to water and oxygen. Thus, catalase prevents the formation of the hydroxyl radical, one of the most common and potent free radicals in living organisms, from hydrogen peroxide through the Fenton reaction [1]. Interestingly, OMWW-induced increase in catalase activity was both time- and dose-dependent suggesting a major role of this enzyme for OMWW's antioxidant effects. Hamden et al. [28] have demonstrated that OMWW extract increased catalase activity in rat plasma, liver, and kidney. Moreover, hydroxytyrosol, one of the main polyphenols present in OMWW, has been shown to increase catalase activity as well as mRNA and protein expression through phosphorylation of AMP-activated protein kinase (AMPK) leading to activation of FOXO3a transcription factor in porcine pulmonary artery endothelial cells [29].

Apart from the catalase activity, the effects of OMWW on SOD activity, an antioxidant enzyme that catalyzes the dismutation of the superoxide anion into hydrogen peroxide and molecular oxygen, in plasma, were examined. The results showed that the water supplied with OMWW decreased SOD activity, especially at 25 days treatment. Other studies have also reported that administration of olive oil polyphenols decreased SOD activity in human and rat plasma [30, 31]. It has been suggested that olive oil polyphenols such as hydroxytyrosol and tyrosol reduce SOD activity by acting as direct scavengers of superoxide anion, that is, it is a kind of compensation mechanism [32, 33]. In contrast, Tufarelli et al. [34] have demonstrated that extra virgin olive oil rich in polyphenols increased SOD activity in chicken liver. Likewise, olive oil polyphenols have been shown to increase SOD activity in rat liver and heart [35, 36]. It seems that the effect of olive oil polyphenols on SOD activity may be tissue specific. In addition, Pajovic et al. [37] have reported that the administration of olive oil to rats affected differently cytosol superoxide dismutase (CuZnSOD) and mitochondrial superoxide dismutase (MnSOD) even in the same tissue.

GSH in erythrocytes was another important antioxidant molecule that was increased after the administration of polyphenols from OMWW through water supply. However, OMWW's effect on GSH was peculiar, that is, there was only significant increase after administration of the low dose (i.e., 20 *μ*g/ml of polyphenols) of OMWW in the chickens at the younger age (after 25 days treatment or 40 days post birth). This finding was in accordance with our previous one observed after the administration of OMWW to chickens through feeding [7]. In this study, feed supplemented with OMWW increased also GSH levels in chickens only at a younger age [7]. As we and others have stated previously, an explanation for this effect may be that polyphenols from OMWW increase GSH at a younger age when the endogenous GSH levels are low, but they had no effect or even reduced GSH in broilers at an older age when chickens' organism can produce by itself efficient GSH [5, 7, 38]. The molecular mechanisms accounting for polyphenols from OMWW-induced increase in GSH levels may be as follows: (i) increase in enzymes being responsible for GSH synthesis (e.g., g-glutamylcysteine ligase and GSH synthetase) [39], (ii) reserve GSH from reaction with free radicals by their direct scavenging [18], and (iii) increase in glutathione reductase (GR) activity (GR regenerates GSH from GSSG) [40].

The abovementioned enhancement of antioxidant mechanisms after the administration of polyphenols from OMWW through water supply may account for the protection from oxidative stress-induced damage. In particular, protein oxidation in plasma as indicated by CARB was lower in the chicken group drinking water containing polyphenols from OMWW compared to that in the control group. This protection was dose dependent after both 25 and 45 days treatments, indicating that it was more intense in the chicken group receiving the high dose of polyphenols from OMWW. The protection of proteins from oxidative stress-induced damage is important, since protein oxidation can impede protein function or lead to destruction of cellular organelles [1]. Specifically, it has been found that 82 mitochondrial proteins have been damaged in chicken skeletal muscle by oxidative stress induced by heat stress [15, 41].

Apart from protein oxidation, drinking water containing polyphenols from OMWW reduced lipid peroxidation in chicken plasma as shown by decrease in TBARS compared to control. Importantly, like protein oxidation, decrease in lipid peroxidation was dose dependent after 25 days treatment, while it was time dependent at both low and high dose of polyphenols from OMWW. It has been demonstrated that climatic stressors such as high dust and NH_3_ levels and low ambient temperature caused lipid peroxidation in chickens [42, 43]. Decrease in lipid peroxidation is considerable in chicken farming, since oxidation of lipids has been associated with lower food intake and egg production [44]. Interestingly, polyphenols such as hydroxytyrosol, verbascoside, and isoverbascoside found in OMWW have been shown to reduce lipid peroxidation [45, 46].

Two different doses, 20 and 50 *μ*g/ml of polyphenols from OMWW, were used in the present study. All the tested oxidative stress markers, apart from GSH, suggested that the high dose of the polyphenolic powder was more effective for improving chickens' redox status. Another interesting finding was that the chickens of control groups at a younger age had higher oxidative stress (e.g., CARB and TBARS) and lower antioxidant mechanisms (e.g., GSH and catalase activity) than the older chickens. This conforms to our observations from previous studies in chickens and lambs [7, 47]. The high sensitivity to oxidative stress of chickens at a younger age emphasizes the need for their antioxidant supplementation in order to prevent pathological conditions.

The bioactive compounds being responsible for the abovementioned antioxidant effects in chickens, drinking water supplied with OMWW, were probably the polyphenols which are known for their antioxidant activity [9, 10]. The chemical analysis of Medoliva powder showed that it was rich in polyphenols, since TPC was the 10% *w*/*w* of the powder. Moreover, although the hydroxytyrosol levels (349.5 pg/ml) in plasma were low, it was shown that it can be absorbed by chickens' organism. Since this is the first study assessing the bioavailability of olive oil polyphenols in chickens, it is not possible to be compared with the other ones. However, studies on the bioavailability of olive oil polyphenols in human have also shown that polyphenols in their free forms present too low levels in plasma or urine due basically to the phase I/II xenobiotic metabolism [48]. Because of these low levels, the polyphenols' ability to exert bioactivities has been questioned. However, it has been suggested that (i) the metabolites derived from polyphenols' metabolism may also be bioactive and (ii) polyphenols may be freed from their conjugates intracellularly [48]. Finally, it should be taken into account that individual polyphenols may present in low levels but their bioactivities are usually attributed to synergistic effects between many different polyphenols [49].

## 5. Conclusions

This is the first study showing that supplementation of broiler chickens with polyphenols from OMWW through drinking water is an easy, cost-effective, and time-saving method for the enhancement of their antioxidant mechanisms (i.e., catalase activity, GSH, and TAC levels) and reduction of oxidative stress-induced damage (i.e., protein oxidation and lipid peroxidation). These findings present particular interest, since different diseases of farm animals have been associated with oxidative stress [3]. The most potent dose was that of 500 *μ*g/ml powder (or 50 *μ*g/ml polyphenols) from OMWW. It should also be taken into account that the exploitation of OMWW for developing high-added value products for animal supplementation is a solution for the environmental problems caused by OMWW.

## Figures and Tables

**Table 1:** Polyphenolic composition and total polyphenolic content (TPC) of Medoliva powder.

Polyphenols	
Hydroxytyrosol	0.50^a^
Tyrosol	0.55
Caffeic acid	0.02
p-Coumaric acid	0.04
TPC	100.00

^a^All values are mg/g powder. TPC: total polyphenolic content (as mg gallic acid/g powder).

**Table 2:** Water consumption by the chickens during the experiment.

	Group A (control)	Group B	Group C
15–40 d after birth	229±67^a^	225 ± 18	233 ± 24
41–60 d after birth	362 ± 12	355 ± 21	358 ± 19

^a^Water consumption (ml) by each chicken per day. Values indicate mean ± SD. ^∗^*p* < 0.05, significant differences from the control (there was not any significant difference between groups).

**Table 3:** Quantification of hydroxytyrosol in chickens' plasma.

Experimental groups	Hydroxytyrosol (pg/ml)
Group A (control)	<LLOD
Group B	349.5
Group C	<LLOQ

LLOD: lower limit of detection (30 pg/ml); LLOQ: lower limit of quantitation (100 pg/ml).
